# Pegaptanib Sodium versus Pegaptanib Sodium Combined with Macular Laser Photocoagulation or Laser Alone for Diabetic Macular Edema

**DOI:** 10.1155/2009/672178

**Published:** 2010-03-09

**Authors:** G. Querques, A. V. Bux, A. R. Fusco, C. Iaculli, N. Delle Noci

**Affiliations:** ^1^Department of Ophthalmology, Ospedali Riuniti, University of Foggia, Viale Pinto, 1 71100, Foggia, Italy; ^2^Department of Hygiene, Policlinico di Bari, University of Bari, 70127 Bari, Italy

## Abstract

*Purpose*. To report the outcomes after primary intravitreal pegaptanib sodium in patients with diabetic macular edema (DME). *Methods*. We conduced a retrospective analysis of eyes with DME treated with primary intravitreal pegaptanib sodium (Macugen) (intravitreal pegaptanib group). The results were compared with the ones of eyes treated with intravitreal pegaptanib sodium associated with macular laser photocoagulation (combined treatment group), and the ones of eyes treated with primary macular laser photocoagulation (macular laser photocoagulation group). *Results*. For the intravitreal pegaptanib group (13 eyes), we found significant changes in mean best-corrected visual acuity (BCVA) and reductions in mean central macular thickness (CMT) at the last follow-up visit (*P* = .0014 and *P* = .0001). For the macular laser photocoagulation group (15 eyes), we found no statistically significant changes in mean BCVA and CMT at the last follow-up visit (*P* > .05). For the combined treatment group (12 eyes), we found no significant changes in mean BCVA at the last follow-up visit (*P* > .05) despite significant reductions in mean CMT (*P* = .0188). *Conclusion*. Intravitreal pegaptanib treatment alone may be superior to macular laser photocoagulation alone and to combined intravitreal pegaptanib treatment associated with macular laser photocoagulation in patients with DME.

## 1. Introduction

Diabetic retinopathy represents the leading cause of blindness in the working age population in the developed world. In patients with type 1 diabetes, visual loss is more commonly due to proliferative changes, while in patients with type 2 diabetes, visual loss is more commonly due to macular edema [1]. Diabetic macular edema (DME) results from the exudation and accumulation of extracellular fluid and proteins in the macula [2] owing to structural changes in the endothelium of retinal vasculature that lead to the breakdown of the blood-retina barrier and an increase in vascular permeability [3]. 

 The gold standard of DME treatment is macular laser photocoagulation. The benefit of immediate focal photocoagulation was demonstrated in the Early Treatment Diabetic Retinopathy Study (ETDRS) [4]. Laser photocoagulation reduces moderate visual loss by 50% (from 24% to 12%, 3 years after initiation of treatment), probably by inducing proliferation of both the endothelial cells in retinal capillaries and retinal pigment epithelial cells, thereby improving the efficacies of both inner and outer blood-retinal barriers [5]. However, in the ETDRS, the patients had only minimal visual improvement after laser treatment and <3% of patients had visual improvements of ≥3 lines at 3 years. Moreover, 12% of eyes developed moderate visual loss at 3 years despite treatment and 40% of eyes with retinal thickening involving the central macula had persistent edema at 12 months [6]. Therefore, a more effective treatment modality is still needed. 

 Recently, intravitreal administration of the corticosteroid triamcinolone acetonide [7–11] and antivascular endothelial growth factor (VEGF) agents [12–15] have been suggested as alternative or adjunct treatments for DME. The probable mechanisms of corticosteroid treatment include increased expression of retinal endothelial cell tight-junction proteins, which diminishes vessel leakage by increasing endothelial barrier properties, and inhibitory effects on VEGF expression [16]. VEGF has been implicated as an important factor in the occurrence of vascular permeability in ocular diseases such as DME [17, 18]. 

Pegaptanib sodium (Macugen, Eyetech Pharmaceuticals, Inc. and Pfizer Inc, New York, NY) is a ribonucleic acid aptamer that targets the VEGF165 isoform that is currently approved in a number of countries worldwide for the treatment of neovascular age-related macular degeneration (AMD) [19]. Phase II trial results of pegaptanib in subjects with DME have been reported recently, [12] and it is currently being evaluated in phase III trials for treatment of DME. Bevacizumab (Avastin, Genentech Inc., San Francisco, CA) is a full-length humanized antibody that binds to all isoforms of VEGF and is used successfully in tumor therapy as a systemic drug [20]. Recent studies have demonstrated the usefulness of intravitreal injections of bevacizumab in the reduction of clinically significant DME, macular edema secondary to central retinal vein occlusion, vascular permeability and fibrovascular proliferation in retinal neovascularization secondary to proliferative diabetic retinopathy, and choroidal neovascularization secondary to AMD [21–26]. Ranibizumab (Lucentis, Genentech, Inc., South San Francisco, CA) is a fully humanized monoclonal antiVEGF antibody binding fragment developed specifically for ophthalmic applications. It was designed to bind all biologically active forms of VEGF and has been effectively used in the treatment of neovascular AMD [27, 28]. A recent study has demonstrated the usefulness of intravitreal injections of ranibizumab in the reduction of clinically significant DME [13]. 

 The purpose of this retrospective study was to report the functional (best-corrected visual acuity [BCVA]) and anatomic outcomes resulting from the use of intravitreal pegaptanib sodium as primary (off-label) therapy in patients with DME, and to compare these outcomes with those obtained with the combination of intravitreal pegaptanib sodium and macular laser photocoagulation, or with laser alone.

## 2. Materials and Methods

We conducted a retrospective study of eyes with macular edema due to type 2 diabetes, treated with intravitreal pegaptanib sodium, or intravitreal pegaptanib sodium combined with macular laser photocoagulation, between October 2006 and December 2008, at the Department of Ophthalmology of the University of Foggia. Institutional review board/ethics committee approval and patients' informed consent were obtained for this study. Before administration of pegaptanib, the off-label use of the drug and its potential risks and benefits were discussed extensively with all patients. We also reviewed the data from a homogeneous group of patients (eyes) treated with macular laser photocoagulation alone as primary therapy for clinically significant DME during the same study period. Excluded were patients with macular edema secondary to causes other than diabetic maculopathy, eyes with DME previously treated with intravitreal triamcinolone, eyes with macular ischemia (≥1 disc diameters of capillary closure at the macula on fluorescein angiography), and the presence of an epiretinal membrane or vitreomacular traction syndrome. Patients who had any ocular surgery within 6 months or significant media opacities and those who were followed for less than 6 months were also excluded. Those that had other DME treatments (such as intravitreal triamcinolone or other antiVEGF therapies) during the follow-up period were excluded from the analysis. Patients with a history of uncontrolled hypertension and recent thromboembolic events were usually not candidates for pegaptanib treatment. 

 Each patient underwent a baseline assessment of BCVA measured at 4 m with standard ETDRS charts and an ophthalmic examination that included slit-lamp biomicroscopy. Baseline central retinal characteristics were analyzed through a dilated pupil performed by a retina specialist by fundus biomicroscopy, by assessing central leakage on fluorescein angiography (FA), and by optical coherence tomography (OCT; OCT3 Stratus, Carl Zeiss, Dublin, CA) utilizing 6 diagonal slow 6-mm radial line scans (software version 4.0). The retinal thickness of the 1-mm central retina was obtained using the macular thickness map for calculations. 

Pegaptanib sodium injections were performed by a retinal specialist. The product was a preservative-free ready-to-use sterile solution composed of pegaptanib sodium dissolved in 10  mM sodium phosphate and 0.9% sodium chloride buffer injection. Presented in glass syringes sealed with a bromobutyl rubber plunger stopper, pegaptanib sodium injection had a fixed 27-gauge needle with a rubber needle shield (tip cap) and a rigid plastic outer shield. Each eye was prepared with 5% povidone/iodine solution, draped, and a lid speculum was placed. Eyes were anesthetized with topical anesthetic, and 0.3 mg (0.05 ml) of pegaptanib sodium was injected intravitreally through the inferotemporal pars plana 3.5 or 4 mm posterior to the limbus, for pseudophakic or phakic patients, respectively. After the injection, intraocular pressure and retinal artery perfusion were checked, and patients were instructed to administer topical antibiotics for 5 days. Standard focal and/or modified grid macular laser photocoagulation [4, 5] was performed alone or in combination with pegaptanib injection (within the subsequent 3 months). 

 Patients who underwent pegaptanib injection alone or in combination with macular laser photocoagulation were examined at 1 and 6 weeks after each injection and monthly thereafter. Follow-up was scheduled on the basis of the pharmacokinetic properties of pegaptanib sodium [29] and was at least 6 weeks from the last injection (when pegaptanib sodium was presumed to have been cleared from the vitreous). Patients who underwent macular laser photocoagulation alone were examined at 2 weeks and monthly thereafter. After the initial treatment, each patient underwent assessment of BCVA and ophthalmic examination, which included slit-lamp biomicroscopy, fundus biomicroscopy, OCT and FA. FA was performed at the discretion of the examiner and not at every postinjection evaluation, which was usually every 6 weeks. Patients underwent repeat injections when there was a recurrence of DME, defined as a decrease of BCVA (at least 5 letters) associated with an increase of intraretinal fluid due to macular edema on OCT (at least 50 *μ*m) and/or FA after complete or partial resolution in previous follow-up visits. 

 Statistical calculations were performed using STATA 10 MP for MacOs X and the Epinfo 3.3 software package (CDC, Atlanta). The analysis of variance (ANOVA) test for repeated-measures was used to compare the pretreatment (relative to the day in which the treatment was performed) and posttreatment (relative to at least 6 weeks from the last injection) mean BCVA (converted to the logarithm of the minimum angle of resolution [logMAR]) and the mean central macular thickness (CMT) of the 3 treatment groups. Serial comparisons of pretreatment and posttreatment BCVA and CMT were performed using the paired *t* test. The Spearman coefficient was used in the analysis of correlation between mean CMT and mean BCVA for each group. The chosen level of statistical significance was *P* < .05.

## 3. Results

A total of 40 eyes of 40 patients with mild-to-severe nonproliferative diabetic retinopathy, who were treated for DME at least 6 months before, were included for analysis. 15, 13, and 12 eyes were allocated to the macular laser photocoagulation alone, intravitreal pegaptanib alone, and combined treatment (intravitreal pegaptanib and macular laser photocoagulation) groups, respectively. Patient baseline characteristics are summarized in Table 1. 

Repeat therapy was provided to 10/13 (76.9%) eyes in the intravitreal pegaptanib group and in 9/12 (75%) eyes in the combined treatment group. A total of 27 pegaptanib injections were administered to the 13 eyes in the intravitreal pegaptanib group during a mean follow-up period of 6.4 months, and a total of 25 pegaptanib injections were administered to the 12 eyes in the combined treatment group, during a mean follow-up period of 11.9 months. A significantly lower number of injections per month were performed in the combined treatment group compared with the intravitreal pegaptanib group (*P* = .0041). 

 The changes in mean BCVA and CMT for each group are summarized in Table 2. Comparison of the pretreatment and posttreatment data (CMT and BCVA) between the 3 treatment groups was performed considering, as pretreatment values, the data relative to the day in which the treatment was performed, and as posttreatment values the data relative to the last follow-up visit. The mean change in BCVA and CMT of the intravitreal pegaptanib treated group was −0.16 (SD = ±0.15 95%  CI = −0.25, −0.08) and −146.77 *μ*m (SD = ±93.9 95%  CI = −199.4, −94.1). The mean change in BCVA and CMT of the combined treatment group was −0.06 (SD = ±0.14 95%  CI = −0.025, −0.13) and −71.67 *μ*m (SD = ±105 95%  CI = −133, 10.3). The mean change in BCVA and CMT for the macular laser photocoagulation group was 0.03 (SD = ±0.14 95%  CI = −0.04, 0.09) and −19.2 *μ*m (SD = ±54.2 95%  CI = −47.5, 9.1). For the macular laser photocoagulation group alone and for the combined treatment group, there were no significant changes in mean BCVA compared with baseline at the last follow-up visit (*P* > .05). For the intravitreal pegaptanib group, there was a significant improvement in mean BCVA compared with baseline at the last follow-up visit (*P* = .0014). 

 For the macular laser photocoagulation group, there were no statistically significant reductions in mean CMT compared with baseline at the last follow-up visit (*P* > .05). For the combined treatment (intravitreal pegaptanib associated with macular laser photocoagulation) group and for the intravitreal pegaptanib group there were statistically significant reductions in mean CMT compared with baseline at the last follow-up visit (*P* = .0188 and *P* = .0001, resp.). 

For the macular laser photocoagulation group and the intravitreal pegaptanib group, BCVA at last follow-up visit showed a modest but statistically significant inverse correlation with CMT (*r* = −0.52, *P* = .0452, and, *r* = −0.57, *P* = .0428, resp.). Conversely, for the combined treatment group BCVA at the last follow-up visit did not show a statistically significant correlation with CMT (*r* = −0.33, *P* > .05). 

 There were no episodes of inflammation or severe decrease of vision immediately after an injection. During the study, no ocular or systemic adverse events such as thromboembolic events (cerebrovascular accidents, transient ischemic attacks, myocardial infarctions, or peripheral vascular disease) were reported in any of the treatment groups.

## 4. Discussion

In this retrospective study of patients with DME treated at our institution, intravitreal pegaptanib was found to produce significant improvements in mean BCVA and reductions in mean CMT from baseline, while with laser photocoagulation alone no significant changes in either measure were apparent. Interestingly, with combined treatment the change in mean BCVA from baseline to final assessment was not significant despite a significant reduction in mean CMT from baseline; moreover, for this group BCVA at the last follow-up visit did not show a significant correlation with CMT (*r* = −0.33, *P* > .3025). Therefore, we can infer that in our study intravitreal pegaptanib treatment alone was superior to combined intravitreal pegaptanib and macular laser photocoagulation treatment, and to macular laser photocoagulation alone, in regard to functional (BCVA), and both anatomical (CMT) and functional (BCVA) results, respectively. Based on the finding that, for the combined treatment group, despite a good anatomical response a similar functional result was not so obvious, one can speculate that macular laser photocoagulation-related sides effects such as paracentral scotoma, subretinal fibrosis, and inadvertent foveolar burns [30, 31] could be responsible for the absence of significant changes in mean BCVA (which was observed in the macular laser photocoagulation group, as well) despite significant reductions in mean CMT, probably antiVEGF therapy-related (which was not observed in the macular laser photocoagulation group). Alternatively, such discrepancy could be due to irreversible degenerative macular changes in this group (which, owing to the small sample size, may have represented a significant bias). In fact, there are many variables including atrophy or destruction of the photoreceptors or retinal pigment epithelium, decreased photoreceptor function, and altered signal processing within the macula that may preclude BCVA benefits despite the presence of measurable alterations in CMT. 

 The treatments used in this study appeared to be safe. No ocular or systemic adverse events such as thromboembolic events were reported in any treatment groups during the study period. 

 The Macugen Diabetic Retinopathy Study Group [12] reported gains in VA of ≥10 letters in 34% and ≥15 letters in 18% of patients with DME after intravitreal pegaptanib sodium injections in a randomized, double-masked, multicenter trial with a follow-up of 36 months. 

 Recently, Chun et al. [13] reported that ranibizumab therapy has the potential to maintain or improve BCVA and reduce retinal thickness in patients with DME. In addition, a trial conducted by the Diabetic Retinopathy Clinical Research Network demonstrated that the use of intravitreal bevacizumab in various protocols could reduce DME in some eyes [26] while Arevalo et al. [25] reported that intravitreal bevacizumab at doses of 1.25 or 2.5 mg seemed to provide stability or improvement in BCVA and CMT in patients with DME at 6 months. 

 Considering the key role of VEGF in the pathophysiology of diabetic retinopathy, VEGF blockade is an attractive therapeutic approach. It is possible to block all VEGF isoforms using bevacizumab or ranibizumab. On the other hand, there is evidence to support selective blockade of the VEGF165 isoform as a way to reduce the VEGF-mediated pathologic effect while preserving VEGF-mediated normal physiologic functions [12]. In the eye, VEGF is necessary and critical for normal neuronal and vessel maintenance and performance. VEGF165 is an isoform that is particularly potent in promoting the increases in ocular neovascularization and vessel permeability characteristic of diabetic eye disease [15]. Preclinical studies demonstrate that selective antiVEGF therapy can inhibit pathological ocular neovascularization while leaving physiological neovascularization unimpaired [15]. In contrast, nonselective VEGF blockade has been shown to impair VEGF-mediated normal physiologic functions, causing regression of normal vasculature as well as reduction of VEGF-mediated neuroprotection [32, 33]. Hence, a nonselective VEGF inhibitor that blocks all VEGF isoforms could be more deleterious to retinal function over the long term than a more selective VEGF antagonist that could spare several of the smaller soluble VEGF isoforms within the eye. 

 The specific inhibition of VEGF165 in our study was accomplished with pegaptanib sodium, a ribonucleic acid aptamer. The specificity, high affinity, and relative lack of immunogenicity of aptamers compare favorably with both small and large molecules, including biologic agents such as antibodies and antibody fragments. 

 Our study has several limitations in that it is short term, nonrandomized, uncontrolled, and retrospective, which preclude any estimation of the long-term efficacy or safety of intravitreal pegaptanib sodium. In addition, apart from the small number of patients for each group, another limitation of our study was the lack of standardization of intravitreal pegaptanib injection and macular laser photocoagulation timing. The duration of follow-up of at least 6 months after treatment was also short; however, our findings provide useful comparisons with the 6-month results from other studies. 

 In conclusion, our findings demonstrate that selective inhibition of VEGF165, the VEGF isoform most associated with both pathological ocular neovascularization and increased retinal vascular permeability in diabetic retinopathy, may produce a clinically meaningful and statistically significant benefit in the treatment of DME. Moreover, overall outcomes suggest that intravitreal pegaptanib treatment alone may be superior to macular laser photocoagulation alone and to combined intravitreal pegaptanib treatment with macular laser photocoagulation in patients affected with DME.

## Figures and Tables

**Table 1:** Patients' baseline characteristics. ETDRS: early treatment diabetic retinopathy study, DME: diabetic macular edema, CMT: central macular thickness, BCVA: best corrected visual acuity.

	Pegaptanib Sodium injection group	Combined treatment group	Macular Laser Photocoagulation group
Total N° of eyes	13	12	15
N° of Male patients	8	7	10
Mean age	65,8 ± 6,1 (95% CI = 62.3, 69.2)	62,4 ± 5,5 (95% CI = 59.2, 65.6)	67 ± 8.6 (95% CI = 62.6, 71.6)
Duration of diabetes	13.7 yrs (mean)	12,8 yrs (mean)	14,9 yrs (mean)
Diffuse DME	7 eyes	7 eyes	9 eyes
Cystoid DME	6 eyes	5 eyes	6 eyes
Mean CMT (*μ*m)	509 ± 125,6 (95% CI = 438.6, 579.4)	420,3 ± 116,3 (95% CI = 352.3, 488.2)	335 ± 91,1 (95% CI = 287.4, 382.6)
Mean BCVA (LogMAR)	0,7 ± 0,4 (95% CI = 0.5, 1)	0,3 ± 0,3 (95% CI = 0.1, 0.4)	0.3 ± 0.3 (95% CI = 0.1, 0.4)

**Table 2:** Changes in mean BCVA and CMT for each group. FU: follow up, BCVA: best corrected visual acuity, CMT: central macular thickness.

	Pegaptanib Sodium injection group	Combined treatment Group	Macular Laser Photocoagulation group
Mean FU (months)	6.4	11.9	7.3
Total injections	27	25	—
Repeat treatments/ eye	10/13	9/12	—
Mean BCVA (LogMAR)-Last FU	0.5 ± 0.4 (95% CI = 0.2, 0.7)	0.3 ± 0.2 (95% CI = 0.2 − 0.4)	0.3 ± 0.3 (95% CI = 0.16,0.45)
Mean CMT-Last FU	362.2 ± 122.9 (95% CI = 293.3, 431.2)	348.6 ± 68.5 (95% CI = 308.6, 388.6)	315.8 ± 87.2 (95% CI = 270.2, 361.4)
Mean BCVA (LogMAR) change	−0.16 ± 0.15 (95% CI = −0.25, −0.08)	0.06 ± 0.14 (95% CI = −0.025, 0.13)	0.03 ± 0.13 (95% CI = −0.04, 0.09)
Mean CMT change	−146.77 ± 93.9 (95% CI = −199.4, −94.1)	−71.67 ± 105. (95% CI = −133, −10.3)	−19.2 ± 54.2 (95% CI = −47.5, 9.1)
